# Shell Engineering of ITO Nanocrystals via Seed-Mediated Growth and Precursor Crowding for Broadband Visible- to-Infrared Absorption

**DOI:** 10.1021/acs.chemmater.5c02493

**Published:** 2025-12-11

**Authors:** Priyadarshi Ranjan, Luca Rebecchi, Anjana Panangattil Muraleedharan, Lea Pasquale, Luca Leoncino, Rosaria Brescia, Irene Martin, Michele Ghini, Andrea Rubino, Nicola Curreli, Nicolò Petrini, Candido F. Pirri, Ilka Kriegel

**Affiliations:** † 121451 Istituto Italiano di Tecnologia-->, Genova 16163, Italy; ‡ Center for Sustainable Future Technologies—CSFT@ POLITO, Istituto Italiano di Tecnologia, Via Livorno 60, Torino 10144, Italy; § Dipartimento di Scienza Applicata e Tecnologie (DISAT), 19032 Politecnico di Torino, Torino 10129, Italy; ∥ Materials Characterization Facility, Istituto Italiano di Tecnologia, Genova 16163, Italy; ⊥ Electron Microscopy Facility, Istituto Italiano di Tecnologia, Genova 16163, Italy; # Nanochemistry, 381691 Istituto Italiano di Tecnologia, Genova 16163, Italy

## Abstract

Seed-mediated growth strategies provide a powerful platform for constructing complex nanocrystal architectures by enabling controlled shell deposition on preformed cores. Here, we report a modular synthesis of multicomponent metal oxide nanocrystals featuring compositionally modulated core–shell architectures. Specifically, indium tin oxide (ITO) nanocrystals are engineered through sequential shelling combined with tunable precursor crowding—a strategy analogous to molecular crowding—that reinforces the local precursor concentration near the nanocrystal surface. This environment promotes diffusion-limited growth and enables anisotropic shell formation along with controlled incorporation of otherwise inaccessible transition metal dopants. Undoped shells transform from quasi-spherical to faceted cuboidal morphologies via directional crystallization, while codoping with Fe and Ni disrupts anisotropic growth and introduces visible-light absorption. Unlike conventional doping strategies, our approach yields broadband-active oxide nanocrystals with spatially controlled composition. These findings establish a versatile class of functional oxide heterostructures with tunable visible-to-NIR absorption.

## Graphical abstract

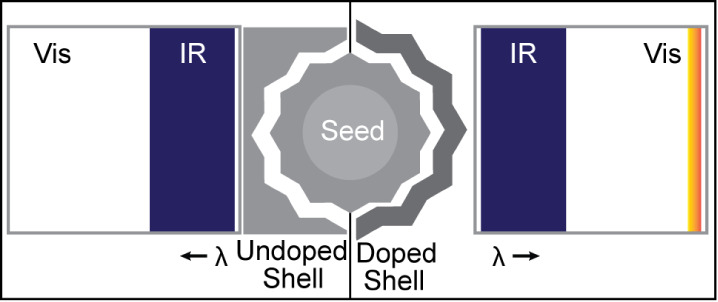

## Introduction

Colloidal nanocrystals serve as versatile building blocks for optoelectronic materials, where morphology, composition, and electronic structure can be precisely engineered to achieve targeted functionality.^1–5^ Among these, indium tin oxide (ITO) nanocrystals are especially appealing for optoelectronic applications due to their high free carrier density, robust plasmonic response, and chemical stability.^6–8^ These properties make ITO nanocrystals promising candidates for applications in infrared photonics, smart windows, sensors, or electrochromic devices.^9–12^

This work builds on three interconnected strategies: (i) seed-mediated growth, in which preformed nanocrystal seeds are reintroduced into fresh precursor solutions;^13,14^ (ii) seed-mediated shelling, which refers to the controlled deposition of outer layers on these seeds;^15^ and (iii) precursor crowding (in analogy to molecular crowding),^16^ where locally concentrated precursors modulate reaction rates and dopant incorporation through diffusion-limited growth. Together, these methods allow us to control both the morphology and composition in complex core–shell metal oxide nanocrystals.

Achieving visible-range optical activity in ITO nanocrystals—while ensuring lattice alignment and tunable synthetic access—remains a key challenge.^17,18^ Although a few semiconductor systems such as HgSe, Te, and doped InP quantum dots display broadband absorption, colloidal metal oxide nanocrystals with engineered, broadband visible–infrared response remain largely unrealized.^19–25^ This gap reflects the synthetic challenges of simultaneously controlling composition, morphology, and dopant distribution in multicomponent oxide nanocrystals.^26,27^ While epitaxial shelling strategies are often pursued to achieve lattice-matched interfaces, their applicability across diverse oxide systems is limited by factors such as lattice mismatch and divergent surface chemistries.^28,29^ Recent studies underscore that even closely related bimetallic oxide systems can yield drastically different outcomes under nominally identical conditions due to differences in precursor speciation, surface ligand coverage, and competing side reactions.^30,31^ These findings highlight the need for tailored synthetic strategies, such as seed-mediated growth and diffusion-limited conditions, to construct compositionally complex oxide architectures.

Doping with transition metals such as Fe and Ni offers a promising route to modulate metal oxide nanocrystal functionality beyond the infrared, enabling midgap states, visible-light absorption, and compositional band structure engineering.^18,24,25,32,33^ Yet, for ITO nanocrystals, the formation of inhomogeneous metal oxide core/shell structures remains largely unrealized.^29^ This gap stems from synthetic challenges such as ligand-specific surface chemistry, precursor–substrate incompatibility, and dopant kinetics, which collectively hinder coherent shell growth in multimetallic oxides.^27^ Recent work by Knecht and Hutchison further demonstrates how amidation-driven activation using oleylamine accelerates monomer generation and promotes facet-selective growth in In_2_O_3_ nanocrystals, stabilizing (100) surfaces even at larger sizes.^31^ Similarly, Plummer et al. show that monomer flux and temperature govern whether nanocrystals adopt branched or faceted morphologies, providing a kinetic framework for shape evolution.^34^ These studies underscore how chemical environment and precursor dynamics critically influence nanocrystal morphology—a principle we exploit through precursor crowding, wherein locally high precursor concentrations in a seed-mediated regime promote anisotropic growth via diffusion-limited conditions, facet-selective reactivity, and suppressed secondary nucleation.^35–37^

Here, we introduce a synthetic strategy that integrates seed-mediated shelling with precursor crowding to overcome these challenges and achieve multimetallic oxide nanocrystals with broadband optical absorption, as shown in Scheme 1. By separating nucleation from shell growth and transferring purified ITO seeds into a fresh precursor solution, we create clean reaction conditions—free from byproducts and enriched with hydroxyl-terminated surfaces—where rapid precursor activation promotes controlled shell formation. This controlled environment allows for the anisotropic growth of undoped shells into faceted cuboids and supports compositional tuning through selective doping with Fe and Ni. Importantly, maintaining high local precursor concentrations establishes a crowded reaction environment where rapid surface deposition outpaces diffusion, creating a kinetically confined regime. This suppresses dopant interdiffusion into the core and favors selective shell growth—reminiscent of crowding effects observed in biomineralization systems, where dense local environments guide spatially controlled assembly.^36–38^

**Scheme 1:**
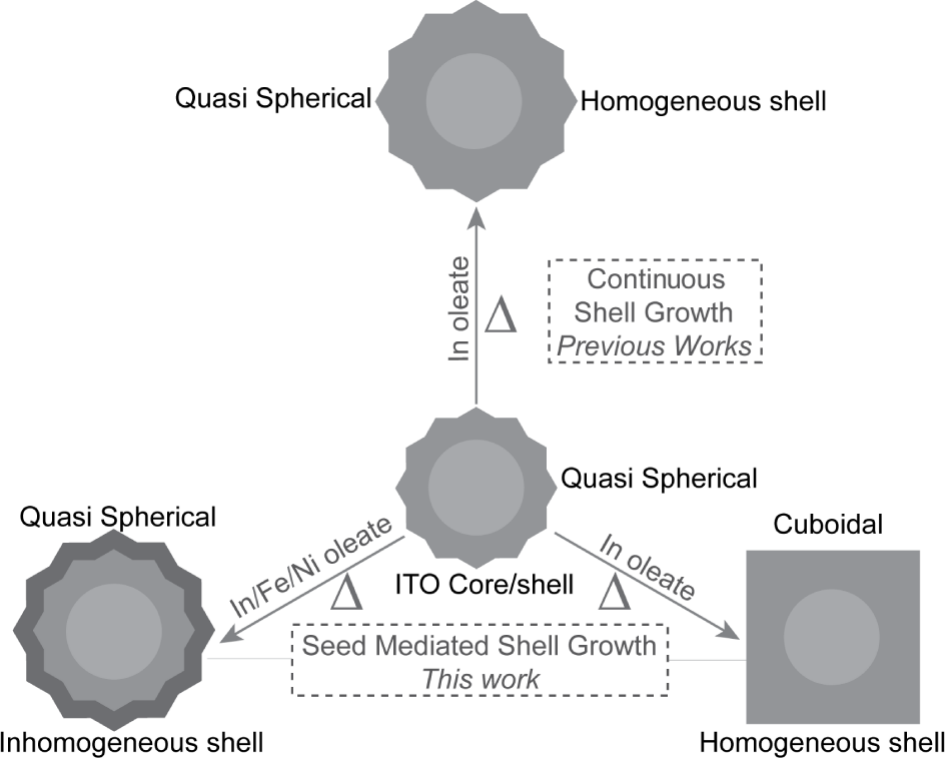
Schematic Representation of Shell Growth in ITO Nanocrystals via Seed-Mediated Strategies and Precursor Crowding, Enabling Simultaneous Morphological and Compositional Control

Our results demonstrate that undoped shells evolve from quasi-spherical to cuboidal morphologies and redshifted infrared plasmonic features. Upon codoping with Fe and Ni, directional growth is disrupted, yielding quasi-spherical nanocrystals with surface-localized strain and visible-light absorption. Elemental mapping reveals dopant-specific spatial segregation, while optical spectroscopy confirms the emergence of additional transitions in the visible regime—resulting in compositionally modulated oxide nanocrystals with a broadband optical response spanning both visible and infrared regions. This platform provides a generalizable route to engineer multifunctional nanomaterials for broadband photodetection, multispectral imaging, and energy harvesting applications.

## Experimental Section

### Materials

Tin­(IV) acetate (CAS: 2800-96-6), indium­(III) acetate (CAS: 25114-58-3), Iron­(III)­acetylacetonate (CAS: 14024-18-1), Nickel­(II) acetate tetrahydrate (CAS: 6018-89-9), oleyl alcohol (technical grade, 85% purity, CAS: 143-28-2), oleic acid (technical grade, 90% purity, CAS: 112-80-1), and toluene (CAS: 108-88-3) were sourced from Sigma-Aldrich. All chemicals were used as received.

### Preparation of Indium Tin Oxide Core/Shell Nanocrystals (1a, 1b, S1 i–iii, 1c)

Samples 1a, 1b, S1 i–iii, 1c, corresponding to ITO core and core/shell nanocrystals, respectively, were synthesized using a continuous injection method adapted from literature.^15,39,40^

For the preparation of (1a, 1b, S1 i–iii, 1c): Briefly, 13 mL of oleyl alcohol was added to a 100 mL three-neck round-bottom flask and heated to 150 °C under stirring and a nitrogen flow for degassing. In a separate 50 mL three-neck round-bottom flask, a mixture of 1 mmol total indium and tin precursors (45 mg of tin­(IV) acetate and 267 mg of In­(III) acetate) and 2 mL of oleic acid was combined, stirred, and heated to 150 °C under nitrogen for 3 h using a Schlenk line to generate the corresponding metal oleates. The temperature of the metal oleates was then lowered to 75 °C, and the stirring oleyl alcohol temperature was raised to 290 °C. An aliquot was taken, constituting the 1a sample. In order to start the shell growth, In­(III) oleate was prepared in a separate flask following the same procedure used for the mixed oleates. Sequential additions of the indium oleate precursor yielded progressively thicker shells, with total added volumes of 0 (1a), 5 (1b), 10 (S1 i), 15 (S1 ii), 20 (S1 iii), and 22.5 mL (1c). In this preparation, every 2 mL of oleic acid corresponds to 291 mg of In­(III) acetate. Aliquots were withdrawn after each defined addition.

After the final addition and subsequent 15 min, heating was discontinued, and the solution was rapidly cooled: first by directing compressed air and spraying acetone onto the flask surface and subsequently using an ice–water bath after reaching ∼160 °C.

The nanocrystals were precipitated by centrifugation at 5540 *g* for 8 min (twice), using ethanol as an antisolvent. The purified NCs were redispersed and stored in toluene for further use.

### Synthesis of Indium Tin Oxide Core/Shell Nanocrystals (1f, 1g, S3 i–iii, 1h)

For the preparation of (1 h): Briefly, 13 mL of oleyl alcohol was added to a 100 mL three-neck round-bottom flask and heated to 150 °C under stirring and nitrogen flow for degassing. In a separate 100 mL three-neck round-bottom flask, a mixture of 1 mmol of total indium and tin precursors (45 mg of tin acetate and 267 mg of In acetate) and 2 mL of oleic acid was combined and heated to 150 °C under nitrogen for 3 h using a Schlenk line to generate the corresponding metal oleates. This precursor dispersion was stirred throughout the degassing period to ensure complete complexation.

Separately, the reaction flask containing oleyl alcohol was maintained at a nitrogen flow rate of 0.130 L/min and heated to 290 °C. The metal oleate solution was cooled to 75 °C, transferred into a nitrogen-purged polypropylene syringe (to avoid syringe deformation), and injected at a rate of 0.3 mL/min using a syringe pump. Within the reaction flask, the metal oleates undergo esterification with oleyl alcohol, producing oleyl oleate and water vapor as byproducts. The water vapor is continuously removed by the nitrogen flow. The reaction was allowed to proceed for 15 min after injection. Hereby, the sample for 1f is prepared.

Separately, to prepare indium oleate for shell growth, 291 mg of indium­(III) acetate and 2 mL of oleic acid were heated at 150 °C for 3 h in a 50 mL three-neck round-bottom flask. This indium oleate solution was injected into the ITO core dispersion (mother solution) at 0.6 mL/min using a syringe pump, while the reaction temperature was raised again to 290 °C and maintained for 15 min. Oleyl oleate, formed as a byproduct, was removed by centrifugation. After 15 min, heating was discontinued, and the solution was rapidly cooled: first by directing compressed air and spraying acetone onto the flask surface and subsequently using an ice-water bath after reaching ∼160 °C.

The nanocrystals were precipitated by centrifugation at 5540 *g* for 8 min (twice), using ethanol as an antisolvent. The purified NCs were redispersed and stored in toluene for further use.

Subsequently, the addition of In­(III) oleate led to the formation of further shells in samples (1g, S4 i−iii, 1h).

### Preparation of Tin Oxide Core/Shell Nanocrystals (Sample 2a)

Sample 2a was synthesized using the same procedure as for 1g but scaled up 4× in volume. The injection rate was increased to 1.2 mL/min.

### Synthesis of 2b and 2c

The ITO core/shell NCs (2a, 1 mmol) were transferred to octane prior to being employed as seeds for this reaction. A 250 mL three-neck round-bottom flask containing 13 mL of oleyl alcohol was heated to 150 °C under nitrogen for 3 h for degassing. Separately, 3 mmol of indium­(III) acetate and 8 mL of oleic acid were combined in a 50 mL three-neck flask and degassed at 150 °C for 3 h.

The flask containing oleyl alcohol was heated to 290 °C under nitrogen (0.130 L/min). The mixed metal oleate precursor solution was cooled to 75 °C and injected into the reaction flask at a rate of 1.2 mL/min using a syringe pump. The growth time was 15 min from the start of the injection. The reaction was then quenched by removing the heat source and cooling the flask, first with a compressed air stream and acetone spray, followed by immersion in an ice-water bath at ∼160 °C.

The nanocrystals were precipitated by centrifugation at 5540 *g* for 8 min (twice) using ethanol as an antisolvent. The purified NCs were redispersed and stored in toluene for further use.

Sample 2c was synthesized analogously using 2b as the seed and 3 equiv of indium oleate. Purification was performed as described above.

### Synthesis of In–Fe–Ni Oxide/Indium Tin Oxide Core/Shell Nanocrystals (4a)

The ITO core/shell NCs (2a, 1 mmol) were transferred to octane prior to being employed as seeds for this reaction. A 250 mL three-neck round-bottom flask containing 13 mL of oleyl alcohol was heated to 150 °C under nitrogen for 3 h for degassing. Separately, a mixture of indium, iron, and nickel precursors in a 0.6:0.3:0.1 molar ratio (total of 3 mmol) and 8 mL of oleic acid was combined in a 50 mL three-neck flask and degassed at 150 °C for 3 h.

The flask containing oleyl alcohol was heated to 305 °C under nitrogen (0.130 L/min). The mixed metal oleate precursor solution was cooled to 75 °C and injected into the reaction flask at 1.2 mL/min using a syringe pump. The growth time was 15 min from the start of injection. The reaction was then quenched by removing the heat source and cooling the flask first with a compressed air stream and acetone spray, followed by immersion in an ice-water bath at ∼160 °C.

The nanocrystals were collected by centrifugation at 7000 *g* for 8 min (twice) using a solvent mixture of *n*-hexane, 2-propanol, and acetone (v/v/v = 1:2:2) as the antisolvent. The final product was redispersed in toluene for storage.

For reaction S10, in a reaction similar to reaction 4a, a mixture of indium and iron precursors in a 0.6:0.4 molar ratio (total 3 mmol) was used instead of a mixture of indium, iron, and nickel precursors in a 0.6:0.3:0.1 molar ratio (total 3 mmol). The rest of the reaction procedure and purification method remained the same as for 4a.

### Materials Characterization

Transmission Electron Microscopy (TEM) was employed to characterize dimensions, morphology, and local crystallinity.

Overview bright-field TEM (BF-TEM) images and selected-area electron diffraction (SAED) patterns were acquired on a JEOL JEM-1400Plus TEM (LaB_6_ thermionic source) operated at 120 kV. SAED patterns provide crystallinity information averaged over a large number of nanocrystals, which are located in a circular area with a 5.2 μm diameter. Azimuthal integration of the SAED pattern, background subtraction and calculation of the powder electron diffraction reference pattern from the ICSD card were performed by means of scikit-ued, a Python package for data analysis and modeling in electron diffraction.^41,42^ The patterns in the manuscript are reported as a function of spatial frequency and scattering angle, with the latter calculated assuming the Kα line of Cu, for ease of comparison with pXRD patterns. High-resolution TEM (HRTEM), energy-filtered TEM (EFTEM), and energy-dispersive X-ray spectroscopy (EDS) analyses were carried out on an image-Cs-corrected JEOL JEM-2200FS TEM, equipped with an in-column image filter (Ω-type) and a Bruker X-Flash 5060 silicon-drift detector system, operated at 200 kV. HRTEM images were acquired using a direct-electron detection camera (K2 Summit, Gatan) at a comparatively low dose rate (about 30 e^–^/(s·Å^2^)) to minimize carbon contamination. The images presented here were extracted from original (280 nm)^2^ frames, obtained by cross-correlated summation of 40 frames, each obtained with a 0.3 s exposure. The HRTEM images were Fourier-filtered, using an average background subtraction filter (ABSF),^43^ to minimize the contribution to the contrast from the amorphous component. The peak pair analysis (PPA)^44^ method was used to compute the mean dilation map from the HRTEM image of individual core–shell particles, with the aim of estimating a possible cell parameter variation in the outer shell.

Quantification of scanning TEM (STEM)-EDS spectra has been obtained for summed spectra acquired from a large number of particles and using the Cliff–Lorimer method for the K peaks of In, Sn, Fe, and Ni.

As STEM-EDS mapping did not provide enough spatial resolution, EFTEM was used to map In, Fe, and Ni distributions over the particles. The elemental maps were obtained by the three-window method at the L_3_ ionization edges of Ni and Fe and at the M_45_ edge of In. Image analysis was performed using Adobe Photoshop software (Version 26.6.0) for the diameter analysis of the particles. The Image analysis was performed by measuring (50–60) nanoparticles. The box fittings of the measurements were obtained from OriginPro 2022 (64-bit) SR1 version 9.9.0.225.

TEM-based particle size distributions were summarized using median ± median absolute deviation (MAD), following best practices for skewed or multimodal data sets in nanoparticle characterization.^45,46^ The MAD metric is significantly less influenced by outliers than the standard deviation, yielding a more robust representation of the central size (or shape) values.

Powder XRD Analysis: XRD patterns of the NCs were collected on a PANalytical Empyrean X-ray diffractometer equipped with a 1.8 kW Cu Kα ceramic X-ray tube, PIXcel3D 2 × 2 area detector, and operating at 45 kV and 40 mA. The samples were prepared by drop-casting a concentrated NC solution onto a zero-diffraction silicon substrate. The diffraction patterns were performed at ambient conditions in a parallel-beam geometry and symmetric reflection mode. High Score 4.1 software from PANalytical was used for phase identification.

Inductively Coupled Plasma–Optical Emission Spectroscopy (ICP-OES): Sn, Fe, and Ni concentrations of the NCs were estimated by ICP-OES on an iCAP 6000 spectrometer (Thermo Scientific). Prior to the measurement, 20 μL of the DMSO-*d*_6_ sample was diluted to 10 mL in an aqueous solution of aqua regia (1:10) and subjected to an acid digestion overnight. Concentration values computed using ICP-OES account for only the inorganic mass of the ITO NCs.

## Results and Discussion

### Morphological Control via Growth Strategy

We began by comparing continuous and seed-mediated growth strategies to investigate their effects on nanocrystal morphology. In the continuous synthesis route, we followed a modified version of the method developed by Hutchison and coworkers.^15,39^ In this approach, ITO cores and their undoped In_2_O_3_ shells were synthesized in a single uninterrupted injection sequence by progressively altering the precursor feed ratio. Specifically, mixed indium–tin oleates were first injected to nucleate and grow Sn-doped In_2_O_3_ (ITO) nanocrystals. Once the desired core size was reached, the precursor composition was changed to omit further Sn addition while maintaining In­(III) oleate injection to promote undoped shell growth. This gradual transition yielded In_2_O_3_/ITO core–shell nanocrystals through a continuous, uninterrupted synthesis process.

Aliquots were taken at regular intervals (Figures 1a–c and S1), capturing samples at various shell thicknesses. The progression from core to core/shell was achieved by sequentially adding increasing volumes of indium oleate, with the final purification carried out through antisolvent precipitation and redispersion. While this method permits size control, bright-field transmission electron microscopy (BF-TEM) analysis (Figures 1a–c and S1) reveals quasi-spherical morphologies with limited facet extension. This is attributed to residual ligands and polymerization byproducts that accumulate during prolonged precursor injection, disrupting anisotropic crystal growth.^31,40^ Inductively coupled plasma–optical emission spectroscopy (ICP-OES) analysis across the shelling series (Figure S2) shows a progressive decrease in Sn content—from 10.19% in the core (1a) to 1.25% in the third shelled sample (S1ii)—consistent with the decreased ratio of the Sn-doped core to the undoped In_2_O_3_ shells. A systematic size increase (Figure 1d) and corresponding redshift in infrared absorption (Figure 1e) are observed, reflecting increased nanocrystal volume, overall lower doping level, and increased shell thickness. The shoulder emerging with shell growth and the redshift of the plasmon peak arise from changes in carrier density distribution, dictated by the spatial arrangement of electrons driving the plasmon resonance. The free carriers, originating from Sn dopants, remain confined to the NC core and are separated from the surface by a depletion region induced by Fermi level pinning. Shell growth alters the surface and thus the carrier distribution. Although the In_2_O_3_ shell is undoped, the core carriers experience a modified dielectric environment, leading to changes in the surface band bending and carrier profile. Optical modeling enables a quantitative description of these electronic rearrangements and their evolution with shell thickness and dopant type (see Figure S3 and Table S1).^15,47,48^ We acknowledge that minor defect formation from oxygen exposure during purification or storage cannot be fully excluded; however, its overall influence is expected to be negligible compared to that of the dominant carrier activation by Sn dopants.

**Figure 1:**
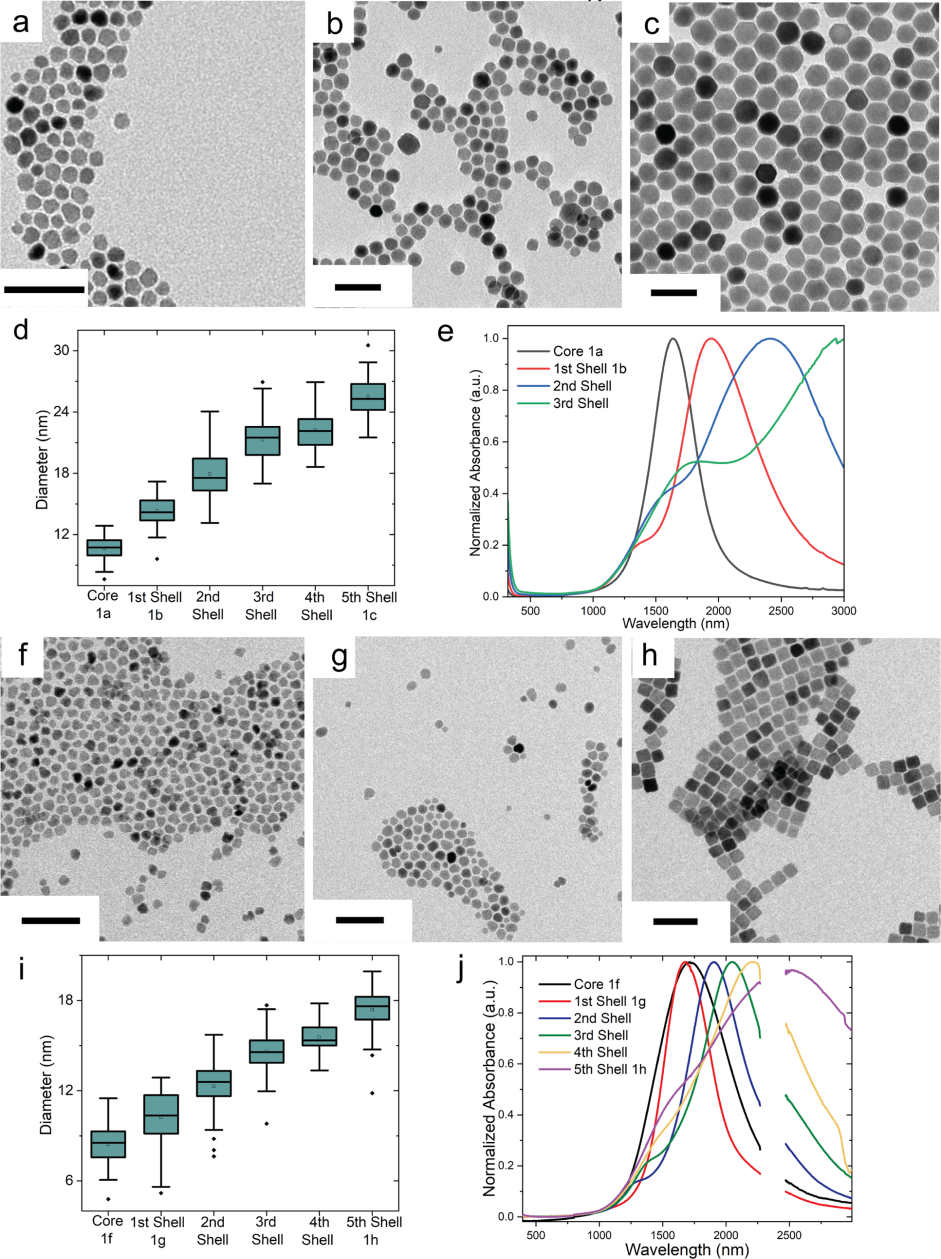
Morphological and optical evolution of ITO cores with indium oxide shell growth via continuous and seed-mediated strategies. (a–c) BF-TEM images of indium tin oxide (ITO) core, first shell, and fifth shell nanocrystals synthesized via continuous growth, respectively, showing progressive size increase. Scale bar: 50 nm. (d) Dimensional analysis of continuously grown nanocrystals; dots represent outliers. (e) UV–vis–NIR absorption spectra showing a red-chromic shift with increasing shell thickness. (f–h) BF-TEM images of ITO core, first shell, and fifth shell nanocrystals synthesized via seed-mediated growth, respectively, revealing controlled shelling and shape evolution. Scale bar: 50 nm. (i) Dimensional analysis of seed-grown nanocrystals. (j) UV–vis–NIR spectra showing a comparable redshift in LSPR with increasing particle size for seed-grown samples.

In contrast, the seed-mediated approach developed by us decouples nucleation from growth by isolating purified In_2_O_3_/ITO core/shell nanocrystals synthesized in the continuous synthesis route and reintroducing them into fresh precursor solutions for successive shelling cycles. Each cycle=comprising injection of the purified nanocrystals, growth, quenching, and purification=promotes shell extension on clean surfaces, free from residual precursors or side products. The BF-TEM images (Figures 1f–h and S4) illustrate a progressive morphological transition from quasi-spherical to cuboidal nanocrystals. Dimensional analysis (Figure 1i) highlights the emergence of shape anisotropy after the third shelling step, while optical spectra (Figure 1j) show a parallel redshift in the IR plasmon band. The particle sizes (median ± median absolute deviation (MAD)) for samples are 8.5 ± 0.8 nm, 10.3 ± 1.2 nm, 12.6 ± 0.8 nm, 14.5 ± 0.7 nm, 15.3 ± 0.7 nm, and 17.3 ± 0.8 nm, respectively. As for the continuous growth samples, the seed-mediated spectra were also analyzed using multilayer optical modeling^48^ to quantify dopant redistribution during shell growth (see Figure S5 and Table S2). A precise interpretation also requires considering NC geometry, shape,^49^ and dispersion effects,^50^ which influence FWHM broadening and resonance modes. The modeling reveals a clear redistribution of carriers, with the active region (core + shell) expanding as the shell grows and the energy-band curvature flattening. This leads to a reduced carrier density in the core (redshifted plasmon peak) and the formation of an active shell region, characterized by its own plasma frequency and damping. Under the constraint of a constant total electron number, the core plasma frequency decreases progressively, while the shell plasma frequency also diminishes with increasing shell thickness due to carrier dilution and the simultaneous growth of the depletion layer. Notably, both continuous and seed-mediated strategies enable systematic shell growth but operate through fundamentally different kinetic regimes. The continuous injection method, in particular, exhibits characteristics reminiscent of living polymerization=where shell extension proceeds in a controlled, monomer-limited fashion as long as reactive species are supplied.^39^ In contrast, the seed-mediated approach enables surface reactivation between discrete shelling steps, allowing for enhanced control over facet development and shape anisotropy.^14,51^ Together, these strategies underscore how nanocrystal shells can be grown with temporal and structural control, akin to polymer chain growth under living conditions.

To probe the effect of local monomer density and diffusion constraints, we tripled the precursor amount and doubled the rate of injection during sequential shelling. This elevated concentration accelerates precursor condensation at the nanocrystal surface, promoting directional growth and facilitating the faster emergence of shape anisotropy. BF-TEM analysis (Figure 2a–c) shows a pronounced shape change from spherical seeds (2a) to cuboidal nanocrystals after the first (2b) and second (2c) shelling cycles under crowded precursor conditions, consistent with this enhanced kinetic regime. However, under these conditions, we also observe signs of secondary nucleation, suggesting that excessive precursor concentration can override the nucleation suppression typically enabled by the seed-mediated approach.^52^ Selected area electron diffraction (SAED) patterns (Figure 2d) reveal an increase in the intensity of the (400) reflection with respect to the (222) and (440) peaks. This is in agreement with dominant (100) facet growth and consequent preferential orientation on the support film, as observed by Plummer et al. during low-flux growth of In_2_O_3_, where monomers selectively attach to step edges on low-energy surfaces to yield cubic morphologies.^34^ Dimensional analysis (Figure 2e) confirms particle growth and aspect ratio evolution, while UV–vis–NIR spectra (Figure 2f) show further redshift samples 2b and 2c compared to sample 2a, highlighting larger shell growth.^47^ As in the previous case, multilayer optical model fitting of the absorption spectra shows a spatial redistribution of carriers with shell growth, causing a decrease in core carrier density and a corresponding redistribution into the shell. The resulting enlargement of the active region aligns with the reduced energy-band steepness but does not increase proportionally with shell thickness (see Figure S6 and Table S3). These findings underscore the synergistic role of surface reactivity and precursor crowding in directing anisotropic shell formation. The particle sizes (median ± MAD) for samples 2a–2c are 9.5 ± 1.1 nm, 10.5 ± 0.9 nm, and 12.3 ± 1.9 nm, respectively.

**Figure 2:**
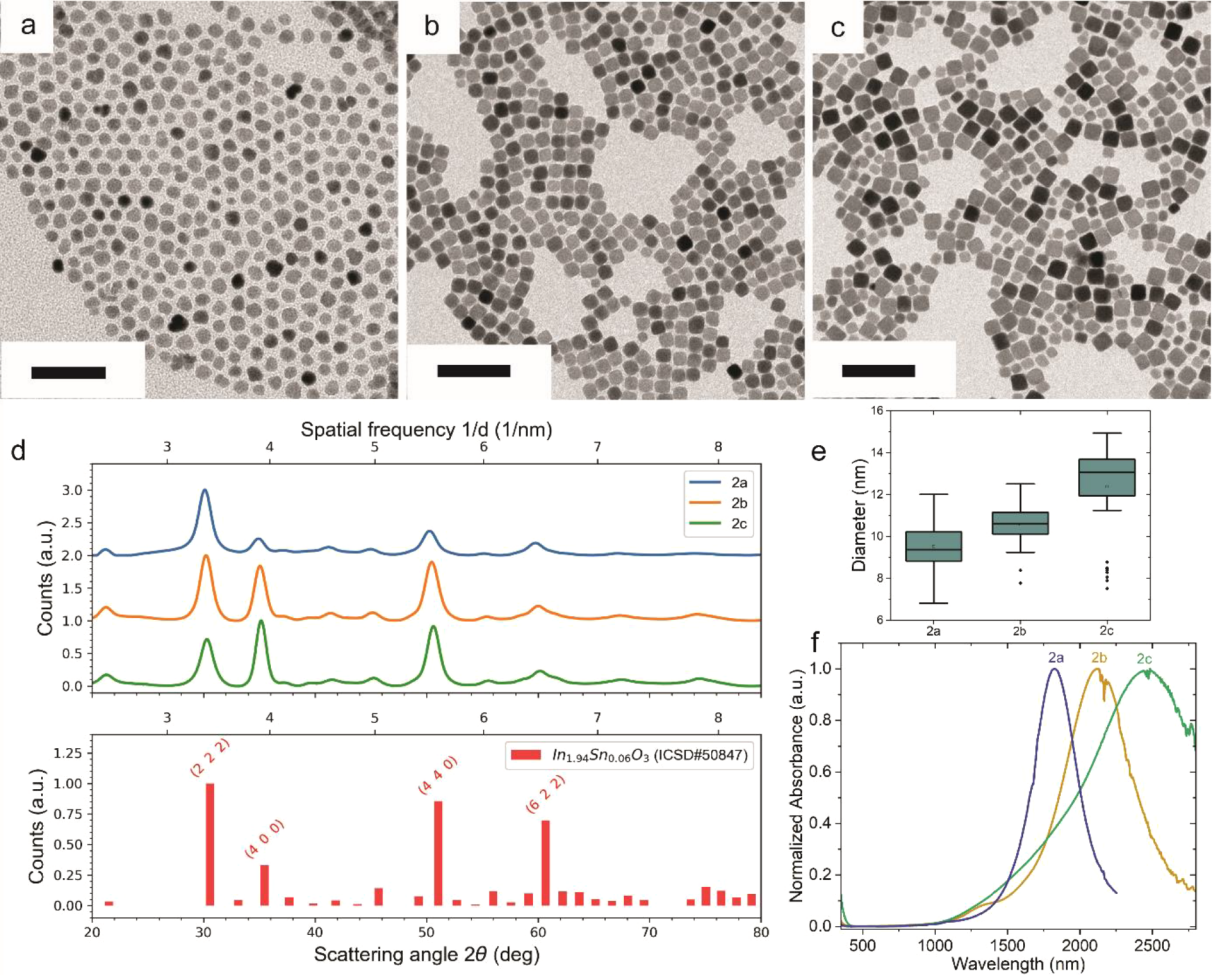
Morphological and structural evolution of In_2_O_3_/ITO nanocrystals grown via a seed-mediated strategy highlighting the effect of precursor crowding. BF-TEM images: (a) Seed nanocrystals (2a) and (b,c) core/shell cuboidal nanocrystals after sequential growth steps. Scale bar: 50 nm. (d) Azimuthally integrated SAED patterns for samples 2a (blue), 2b (orange), and 2c (green), overlaid with powder electron diffraction peaks for the ITO reference (red bars; ICSD 98-005-0847). Intensification of the (400) peak and decrease of the (222) and (440) peaks confirm anisotropy toward a cuboidal shape. (e) Dimensional analysis of nanocrystals showing particle size increase and shape evolution. (f) UV–vis–NIR absorption spectra of ITO nanocrystals: 2a (blue), 2b (orange), and 2c (green). A progressive redshift is observed with increasing nanocrystal size.

These contrasting outcomes can be rationalized by differences in precursor activation kinetics and surface chemistry. In continuous growth, the relatively slow esterification between metal oleates and oleyl alcohol delays monomer generation, reducing the likelihood of directional growth and limiting the emergence of well-defined shape asymmetry. These findings are consistent with the model proposed by Jansons and Hutchison, where facet-selective growth in indium oxide reflects a balance between surface energy and hydroxide coordination: high-energy (001) facets dominate at early stages but are gradually replaced by more stable (011) and (012) planes during extended growth, yielding cuboctahedral shapes.^15^ Moreover, residual ligands and polymerization byproducts in continuous growth further inhibit ordered deposition. Complementary insights from Plummer et al. underscore the critical role of monomer flux in morphology selection: high flux leads to branched nanocrystals due to hindered surface diffusion, whereas low flux promotes faceted, cuboidal growth via layer-by-layer monomer attachment on low-energy (100) facets.^34^

In contrast, the seed-mediated strategy=applied to purified nanocrystal surfaces rich in hydroxyl groups=facilitates rapid precursor activation and efficient surface binding. The absence of residual ligands and nucleation byproducts ensures unimpeded access of reactive monomers to the seed surface, accelerating anisotropic shell growth relative to continuous synthesis. As shelling proceeds, nanocrystals evolve from quasi-spherical to faceted cuboids, reflecting directional crystallization driven by surface reactivation and precursor crowding (Figure 3). This behavior reflects how synthetic history and local monomer availability dictate both shape evolution and the optical response. These results validate prior models of facet-selective growth in nanocrystals while expanding them to incorporate diffusion-controlled precursors as a powerful lever for morphological precision.^51^ Notably, a comparison between the sequential shelling shown in Figure 1 (moderate monomer loading) and Figure 2 (high monomer loading and enhanced rate of injection) reveals that enhanced precursor crowding accelerates the emergence of shape anisotropy and preserves it across subsequent shelling steps. This suggests that high local monomer density not only promotes directional growth during initial shell formation but also stabilizes anisotropic morphologies against reversion during continued precursor addition.

**Figure 3:**
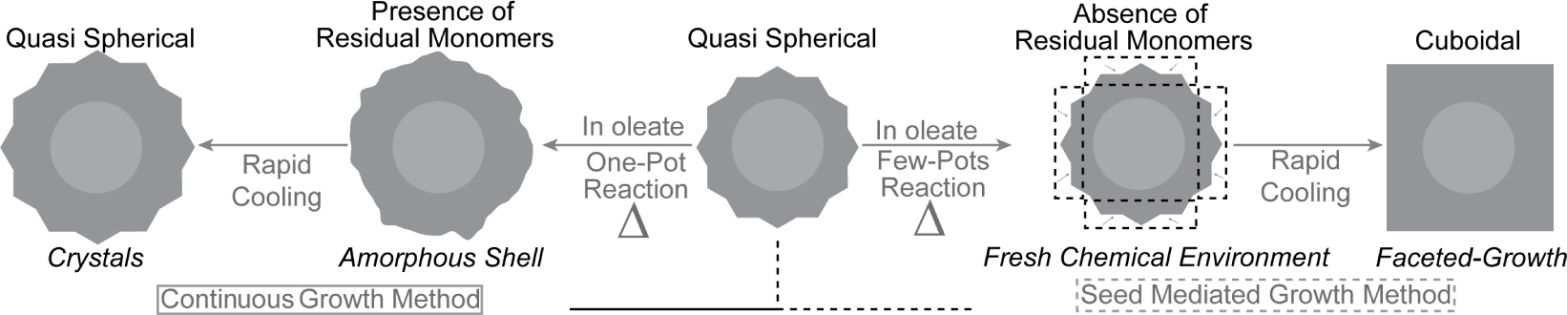
Schematic comparison of nanocrystal growth mechanisms. In the continuous growth method (left), residual surface-bound monomers and reaction byproducts accumulate during heating, delaying the onset of the growth phase and leading to slower and less controlled shell formation. In contrast, seed-mediated growth (right) proceeds under cleaner conditions: purified seed particles are introduced into fresh precursor solutions after rapid cooling (removal of the heating mantle, air quenching, and ice bath immersion at 160 °C) and purification, which remove side products and expose hydroxyl-rich surfaces. These clean conditions enable faster precursor incorporation, promoting more rapid shell growth and improved shape control.

This mirrors the kinetic landscape described by Knecht and Hutchison, where fast amidation leads to early nucleation closure and rapid surface-directed growth on (100) facets.^31^ Their findings support the concept of diffusion-controlled kinetic confinement, where the rate of precursor activation=not just total concentration=shapes the final morphology and dopant incorporation pathways, similar to how our precursor crowding drives anisotropic shell extension.

### Compositional Control through Dopant Incorporation

Having established a robust morphological control strategy via seed-mediated shell growth, we next explored how compositional complexity impacts the nanocrystal structure. Motivated by this insight, we extended this method to explore the formation of inhomogeneous shells, a relatively underexplored domain in ITO systems. Specifically, we introduced transition metal dopants (Fe and Ni) into the shell by employing mixed In/Fe/Ni precursors under otherwise identical seed-mediated conditions (sample 2b). The goal was to examine whether the incorporation of dopants perturbs the observed anisotropic crystallization pathway.

Doping-induced disruption of directional growth manifests as a morphological shift from faceted cuboids to quasi-spherical nanocrystals in sample 4a, as seen in BF-TEM imaging. BF-TEM images (Figure 4a) and size analysis show a median diameter of 9.8 ± 0.7 nm for 4a (Figure 4b). SAED patterns (Figure 4c) as well as a powder X-ray diffraction (pXRD) pattern (Figure S7) reveal a slight peak shift to higher angles, consistent with lattice contraction. High-resolution TEM combined with peak pair analysis further confirms slightly smaller cell parameters in the shell (∼0.3%).

**Figure 4:**
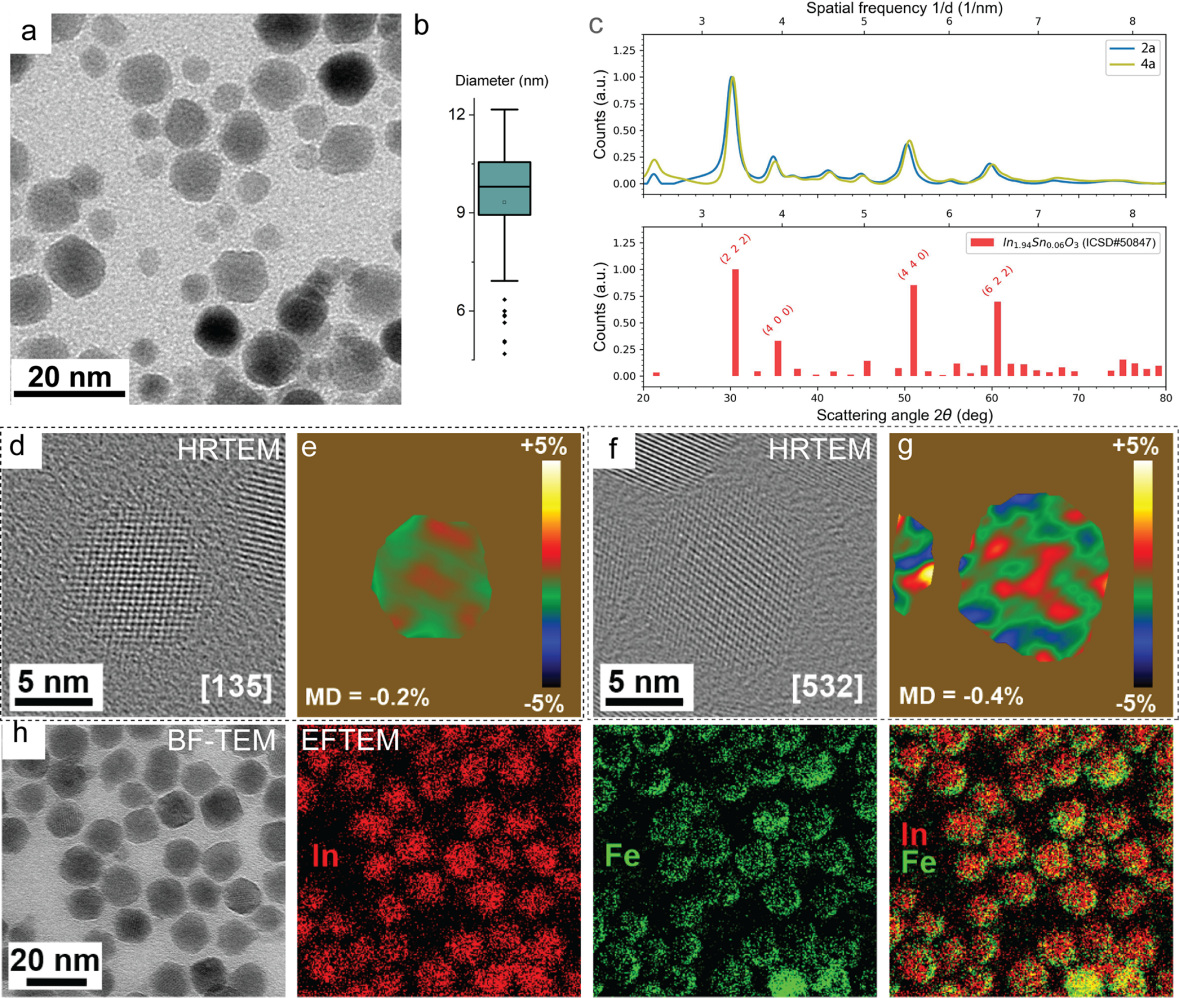
Compositional and structural characterization of Fe/Ni-doped In_2_O_3_/ITO nanocrystals (sample 4a). (a) BF-TEM image showing quasi-spherical morphology for sample 4a. (b) Size distribution plot of 4a. The dots outside the box are outliers. (c) Azimuthally integrated SAED patterns for samples 2a (blue) and 4a (yellow), overlaid with ITO reference data (red bars; ICSD 98-005-0847), showing peak shifts to slightly higher angles and reduced high-angle intensities—indicative of lattice contraction. (d,f) HRTEM images of two particles in sample 4a, with the identification of zone axis. (e, g) Dilation maps from peak pair analysis (PPA) reveal ∼0.3% contraction in the shell, demonstrating smaller cell parameters in the shell material. (h) Elastically filtered BF-TEM image and corresponding maps for In (red) and Fe (green) and their overlap, showing Fe enrichment in the outer shell.

To determine the spatial distribution of Fe and Ni over the particles, we acquired scanning transmission electron microscopy coupled with energy-dispersive X-ray spectroscopy (STEM-EDS) maps (Figure S8) and performed ICP-OES measurements (Figure S9). STEM-EDS elemental mapping does not show any clear localization of Ni and Fe throughout the particles. Energy-filtered TEM (Figure 4h), having an intrinsically higher spatial resolution than STEM-EDS, highlights a higher Fe concentration at the outer shell, while no localized signal is observed for Ni. This contrast in spatial localization implies differing affinities of Fe and Ni for the nanocrystal surface during shell growth. The preferential localization of Fe in the outermost region suggests it is incorporated later during shell formation, possibly due to lower diffusivity or stronger surface binding. Ni, instead, is either incorporated earlier during the growth process or possesses higher diffusivity, allowing uniform integration.^25^

In contrast, shell growth was not observed when Ni was excluded from the precursor mixture, i.e., using only In and Fe precursors under otherwise identical conditions (BF-TEM images, Figure S10 and pXRD pattern in Figure S7). This result underscores the cooperative role of Ni and Fe, alongside In, in enabling shell growth under epitaxial conditions. These observations are consistent with prior studies indicating that dopant incorporation in nanocrystals is not solely dictated by bulk solubility but rather by kinetic surface adsorption processes and facet-specific interactions.^25,53^ The precursor ratios used for codoping (In:Fe:Ni = 0.6:0.3:0.1) were intentionally chosen to exceed the nominal solubility limits of Fe and Ni in bulk In_2_O_3_ in order to access kinetically confined, surface-segregated doping regimes. Under rapid shell growth, a high local precursor concentration promotes transient supersaturation, enabling substitutional dopant incorporation without forming secondary phases.

SAED and pXRD patterns (Figures 4c and S7) confirm that the resulting nanocrystals retain the single-phase cubic In_2_O_3_ lattice, showing no additional reflections associated with Fe- or Ni-oxide phases. STEM-EDS and ICP-OES analyses (Figures S8 and S9) indicate that even under these intentionally concentrated conditions, the overall dopant incorporation remains low (Fe/In = 0.14, Ni/In = 0.087). This observation underscores the kinetic confinement of dopant uptake and is the reason we refer to this regime as precursor crowding, where excess precursors increase local chemical potential but are only partially incorporated into the growing shell. Furthermore, the role of precursor reactivity and differential binding affinities in enabling or suppressing dopant incorporation has been broadly established in colloidal systems, and recent work has shown that the same ligands can play divergent roles depending on the metal ion and nanoparticle surface involved=hindering generalization across systems.^26,27^

Collectively, these results indicate that the seed-mediated process proceeds through kinetically confined layer-by-layer shell growth rather than an alloy-type core expansion. The preserved lattice spacing, sharp diffraction peaks, and shell-localized dopant enrichment confirm heterogeneous deposition at the seed–solution interface under diffusion-limited conditions.

Optical spectroscopy provides further insight. UV–vis spectra (Figure 5a) of Fe/Ni-doped shell nanocrystals (4a) show a distinctly broadened profile compared to undoped shell counterparts (2b), with a pronounced shoulder near ∼480 nm (Figure 5b). This feature is attributed to localized states or transitions induced by surface doping. The broadening and redshift of absorption into the visible region enhance the spectral response, extending beyond the typical IR domain of ITO. The strong absorption band in the near-infrared is due to plasmonic resonances arising from the free carriers in the system inserted via the Sn doping in the In_2_O_3_ core, as discussed above.^54,55^ Multilayer optical model fitting of the plasmon absorption spectra shows a more uniform carrier redistribution compared to that of sample 2b. Unlike the latter, which exhibits two distinct plasma regions, this sample displays a gradual carrier spread from the core to the shell, resulting in a lower overall carrier density. Differently from the previously analyzed cases, the depletion layer is slightly reduced, suggesting a modified Fermi-level pinning energy due to changes in surface chemistry induced by Fe/Ni doping (see Figure S11 and Table S4).

**Figure 5:**
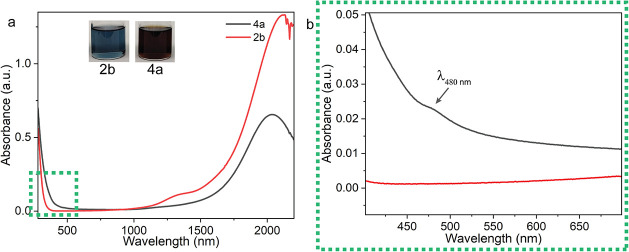
Optical properties of nanocrystal dispersions. (a) UV–vis spectra of 2b (red line; sky blue dispersion in toluene) and 4a (black line; dark brown dispersion in toluene). (b) Expanded inset highlighting a shoulder at ∼480 nm in (a) (black line), possibly associated with intermediate electronic transitions or dopant-induced surface states.

Corroborating the spectroscopic changes, we also observed distinct differences in the color of nanocrystal dispersions: the codoped sample 4a appears dark brown (inset, Figure 5a), 2b exhibits a sky-blue hue (inset, Figure 5a), while nanocrystals synthesized using only Fe and Ni precursors (in the absence of indium) display an olive-green color (Figure S12 inset). Comparable effects have been observed in Fe- and Ni-doped brookite TiO_2_ nanorods.^25^

Mechanistically, Fe’s limited solubility and poor lattice compatibility make it prone to forming separate oxide phases or precipitating unless kinetically trapped during growth. In contrast, Ni exhibits better compatibility and distributes more homogeneously. Drawing inspiration from Zhang et al.‘s strategy for brookite-phase TiO_2_ nanorods, we hypothesize that similar principles apply here: lattice mismatch and precursor reactivity govern dopant behavior.^25^ Under precursor crowding and rapid quenching, Fe localizes at the shell surface, likely due to its stronger surface affinity and lower diffusivity, while Ni=being more mobile=distributes more uniformly. This dynamic is reminiscent of mineralization processes, where confined, crowded environments direct ion incorporation.^38,56^ In our system, crowding via metal precursor mixing mimics such constraints, facilitating dopant retention at targeted locations within the shell.

As summarized in Figure 6, mixed In/Fe/Ni precursors promote dopant (Fe) localization at the interface, whereas Fe-only or Fe/Ni-only systems (i.e., systems lacking indium; Figure S13) fail to form distinct core/shell architectures. We propose that indium oleate plays a dual role=facilitating structural stabilization and mediating selective dopant uptake. The quasi-spherical shape of nanocrystal 4a contrasts with that of the cuboidal 2b, suggesting that dopant diffusion disrupts facet-selective growth. EFTEM mapping supports this view, confirming the spatial inhomogeneity in the shell. Such spatial inhomogeneity in dopant placement enables the controlled tuning of band alignment and interfacial electronic structure. As demonstrated in transition metal-doped TiO_2_ systems, subtle shifts in dopant positioning can strongly influence visible-light absorption and carrier dynamics.^25^ Here, the combination of seed-mediated shelling, dopant crowding, and surface-specific incorporation establishes a modular platform to access tailored optical and electronic properties in complex oxide nanocrystals.

**Figure 6:**
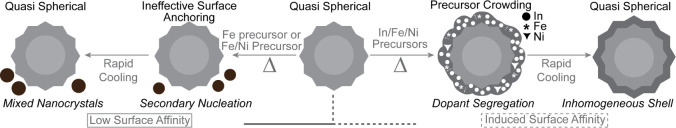
Schematic illustration of nanocrystal formation under varying precursor compositions. Left: Fe or Fe/Ni precursors yielded dispersed mixed nanocrystals. Right: Mixed In/Fe/Ni precursors led to an inhomogeneous shell formation on ITO seeds. The proposed crowding effect, combined with the cointroduction of multiple precursors, limits Fe and Ni diffusion away from the core and enhances their surface trapping during cooling. This behavior reflects induced surface affinity and kinetic confinement, while the failure of Fe or Fe/Ni precursors alone to form shells highlights the role of indium in overcoming ineffective surface anchoring and enabling shell nucleation.

## Conclusion

In summary, we present a modular synthesis of broadband-active metal oxide nanocrystals by coupling seed-mediated growth with precursor crowding. This strategy enables precise morphological control through facet-selective crystallization, while codoping with Fe and Ni introduces dopant-specific spatial asymmetry and visible absorption. By integrating diffusion-limited growth and compositional complexity, this approach offers a generalizable platform for designing multifunctional oxide nanocrystals for visible–infrared optoelectronics, broadband photodetection, and advanced photonic architectures.

## Supplementary Material



## Notes

Theoretical calculations, dimensional analysis, and ICP study for 1a, 1b, S1 i–ii; pXRD patterns of nanocrystals 2a, 4a, and S7; TEM images of control experiments; EDS mapping and spectroscopy of 4a; Elemental ratios of 2a, 2b, 2c, and 4a by ICP-OES; snapshot of a control experiment (PDF)
