# Supplementary material for: Shell Engineering of ITO Nanocrystals via Seed-Mediated Growth and Precursor Crowding for Broadband Visible- to-Infrared Absorption
Source: Chem Mater. 2025 Dec 11;37(24):9932–43. doi: 10.1021/acs.chemmater.5c02493 (PMC12746414; doi:10.1021/acs.chemmater.5c02493)
Supplement: Supplementary file 1 [file cm5c02493_si_001.pdf]

Supporting Information for

Shell Engineering in ITO Nanocrystals via Seed-Mediated Growth and Precursor Crowding for Broadband Visible–Infrared Absorption

Priyadarshi Ranjan [a,b,c]*, Luca Rebecchi [c], Anjana Panangattil Muraleedharan [c], Lea Pasquale [d], Luca Leoncino [e], Rosaria Brescia [e], Irene Martin [c], Michele Ghini [f], Andrea Rubino [c], Nicola Curreli [c], Nicolò Petrini [c], Candido F. Pirri [b,c], Ilka Kriegel [c]*

[a] *Istituto Italiano di Tecnologia; Genova, 16163, Italy*
[b] *Center for Sustainable Future Technologies—CSFT@ POLITO, Istituto Italiano di Tecnologia, Via Livorno 60, Torino 10144, Italy*
[c] *Dipartimento di Scienza Applicata e Tecnologie (DISAT), Politecnico di Torino; Torino, 10129, Italy*
[d] *Materials Characterization Facility, Istituto Italiano di Tecnologia; Genova, 16163, Italy*
[e] *Electron Microscopy Facility, Istituto Italiano di Tecnologia; Genova, 16163, Italy*
[f] *Nanochemistry, Istituto Italiano di Tecnologia; Genova, 16163, Italy*

Multi-layer optical fitting

We analyzed the experimental absorption spectra using multilayer optical modelling, following the fitting procedure described in our previous work.[1] Briefly, the normalized absorption was fitted using a multilayer dielectric function by treating individual NCs as nanospheres of radius R (obtained from TEM measurements) and including an additional surface depletion layer. Core-only and core–shell NCs were modelled using two-layer (2L) and three-layer (3L) approaches, respectively. The detailed formulas are provided in our previous works.[1,2]

The initial dopant concentration (ne_{core}), determined from the core spectra, was fitted using a 2L model consisting of a plasmonic core ($Core$) and a depletion layer (DL) described by a Drude-like dielectric function, including the plasma frequency (ω_{core}) and damping parameter (γ_{core}). To avoid overfitting and reduce multiple solutions, the total number of oscillating electrons (N_{TOTAL}) across the core and core–shell structures was constrained to preserve the total electron count obtained from the core-only case (N_{core}), ensuring charge conservation throughout the series. Under this constraint, the fitting converged to unique parameter sets, providing the shell carrier concentration (ne_{shell}), shell damping (γ_{shell}), shell electrons (N_{shell}) and shell thickness ($Shell$). Derived values of mean free path of carriers in both regions (mfp_{core} and mfp_{shell}) were also calculated. The damping parameter—dependent on both carrier density and the effective electronic extent of the oscillating region—was also monitored to ensure a physically meaningful evolution.

The fitted and experimental spectra are compared in Figures S10–S12, corresponding respectively to Figures 1e (continuous-growth ITO), 1j (seed-mediated ITO), 2f (seed-mediated ITO), and 5a (Fe/Ni–ITO), while the corresponding fitted parameters are respectively reported in Tables S1-S4.

The 3L model successfully reproduces the main spectral features for all cases analyzed, including the red shift of the plasmon peak and the progressive increase of the shoulder intensity with shell growth. However. it should also be noted that, since the different synthesis methods (continuous growth and seed-mediated) can lead to distinct final core–shell geometries (quasi-spherical or cubic), a more refined model accounting for geometry effects should be developed in a dedicated future study in order to take geometrical effects into account in the optical response.[3]

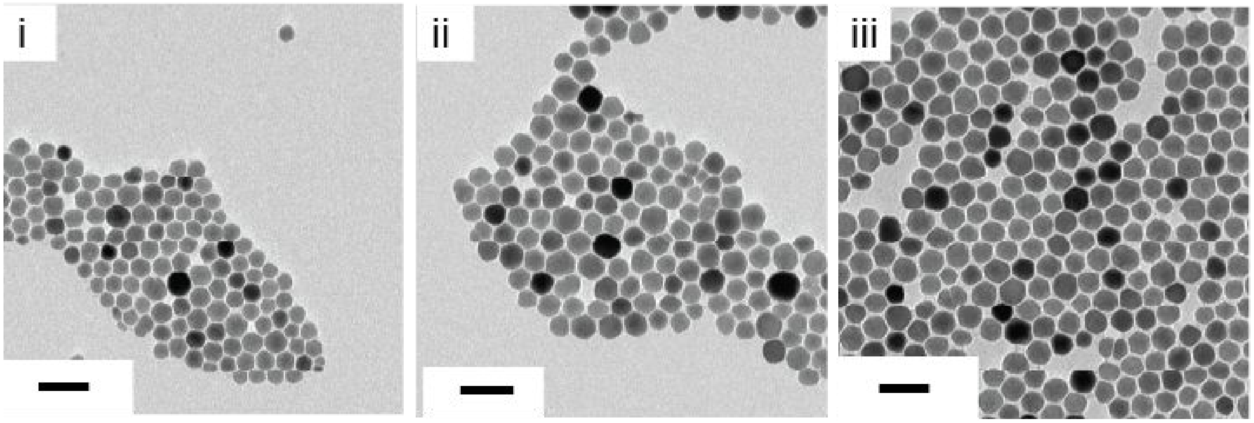

Figure S1. BF-TEM images of indium tin oxide (ITO) nanocrystals after the (i) 2nd, (ii) 3rd, and (iii) 4th shell growth cycles synthesized via continuous growth. A progressive increase in particle size is observed with each successive shell. Scale bar: 50 nm.

Sample	Meadian with median absolute deviation (MAD)	Sn (% mol) by ICP-OES
1a	10.7 ± 0.7	10.19
S1i	14.2 ± 0.9	2.94
S1ii	17.5 ± 1.5	1.64
S1iii	21.5 ± 1.5	1.25

Figure S2. ICP-OES analysis confirming that Sn remains confined to the nanocrystal core during continuous shell growth. Quantified Sn concentrations (% mol) for samples 1a, S1i to iii show a systematic decrease, consistent with progressive dilution of the Sn-doped core by overgrowth of undoped In_2O_3 shells. The drop from 10.19% (1a, core) to 1.25% (S1iii, final shell) indicates that no additional Sn is incorporated during shelling. Based on dilution calculations using $C_i \cdot V_i = C_f \cdot V_f$, the theoretical Sn content expected in the shelling solution (~3.5% for sample 1b) exceeds the measured value (2.94%), further supporting that Sn remains confined to the core and does not leach into or redeposit during shell formation.

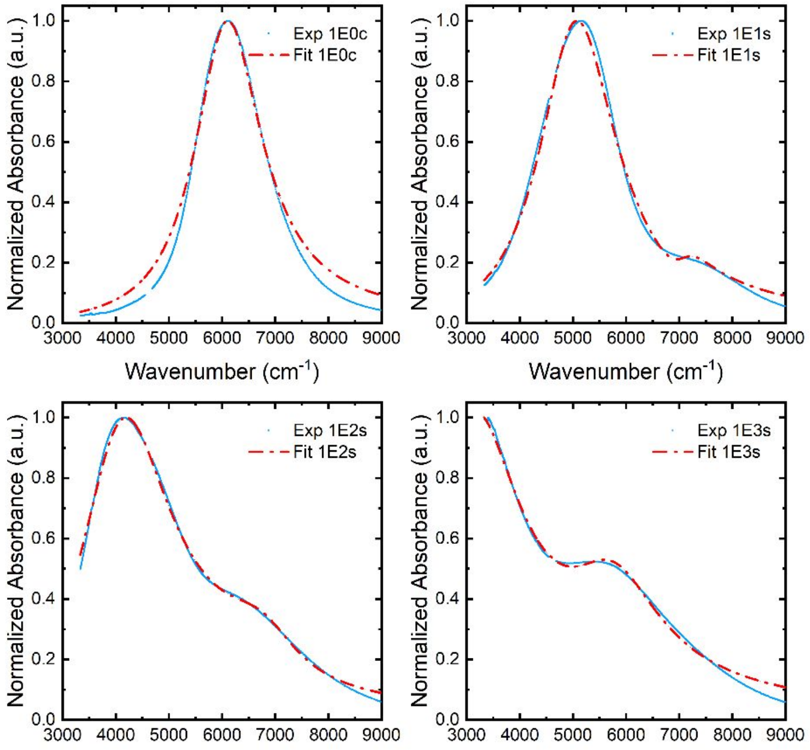

Figure S3. Experimental normalized absorption spectra (blue) and corresponding fitting (dashed-red)) obtained with 2-layer (core case) and 3-layer optical modeling for continuous growth method. Main text corresponding spectra are found in Figure 1e.

Parameters	1EC0	1ES1	1ES2	1ES3
ω_{core} (cm^{-1})	18200.170	17125.954	17343.5729	15991.720
ω_{shell} (cm^{-1})	1.06483	13955.905	12358.005	9206.23614
ne_{core} (m^{-3})	1.477E27	1.308E27	1.342E27	1.141E27
ne_{shell} (m^{-3})	5.057E18	8.687E26	6.812E26	3.780E26
γ_{core}(cm^{-1})	1547.58685	2090.13004	1109.05336	2109.04365
γ_{shell} (cm^{-1})	100000.07	618.97636	2430.77878	2062.52938
mfp_{core}(nm)	3.49974	2.4883	4.72912	2.35587
mfp_{shell} (nm)	8.16258E-5	7.33057	1.72131	1.6671
N_{TOTAL}	688.88289	679.93684	650.61131	639.80716
N_{core}	688.88289	595.82144	288.40766	336.99051
N_{shell}	1.79752E-6	84.1154	362.20365	302.81665
R (nm)	5.05	7.2	8.05	9.8
$Core$ (nm)	4.81038	4.77291	3.71613	4.13165
$Shell$ (nm)	0	0.31675	1.91182	2.26523

$DL\ (nm)$	0.23962	2.11034	2.42205	3.40312

Table S1. Fitting parameters corresponding to core (C0) and shell (S1, S2, S3) samples obtained via continuous growth synthesis method corresponding to Figure 1e.

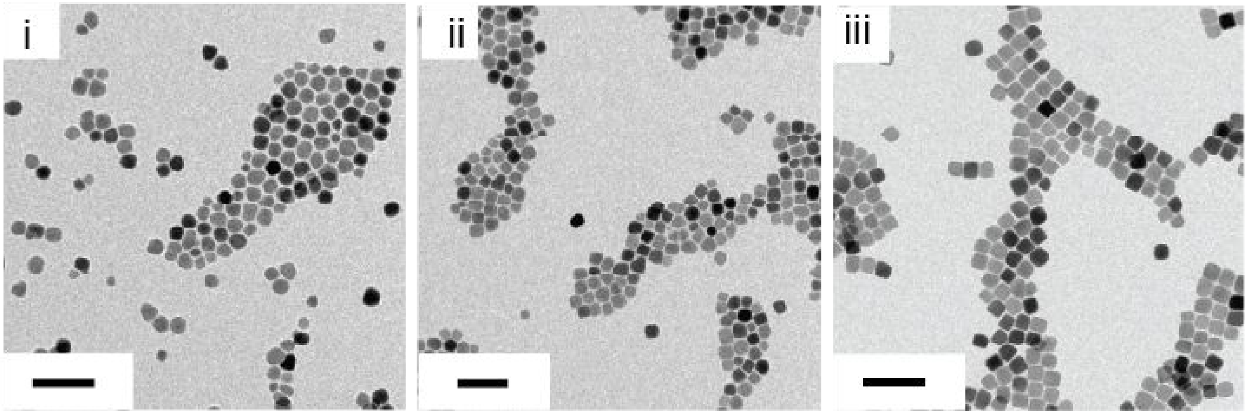

Figure S4. BF-TEM images of ITO nanocrystals after the (i) 2nd, (ii) 3rd, and (iii) 4th shell growth cycles synthesized via seed-mediated growth. A progressive increase in size is observed, with shape anisotropy emerging after the third shell cycle. Scale bar: 50 nm.

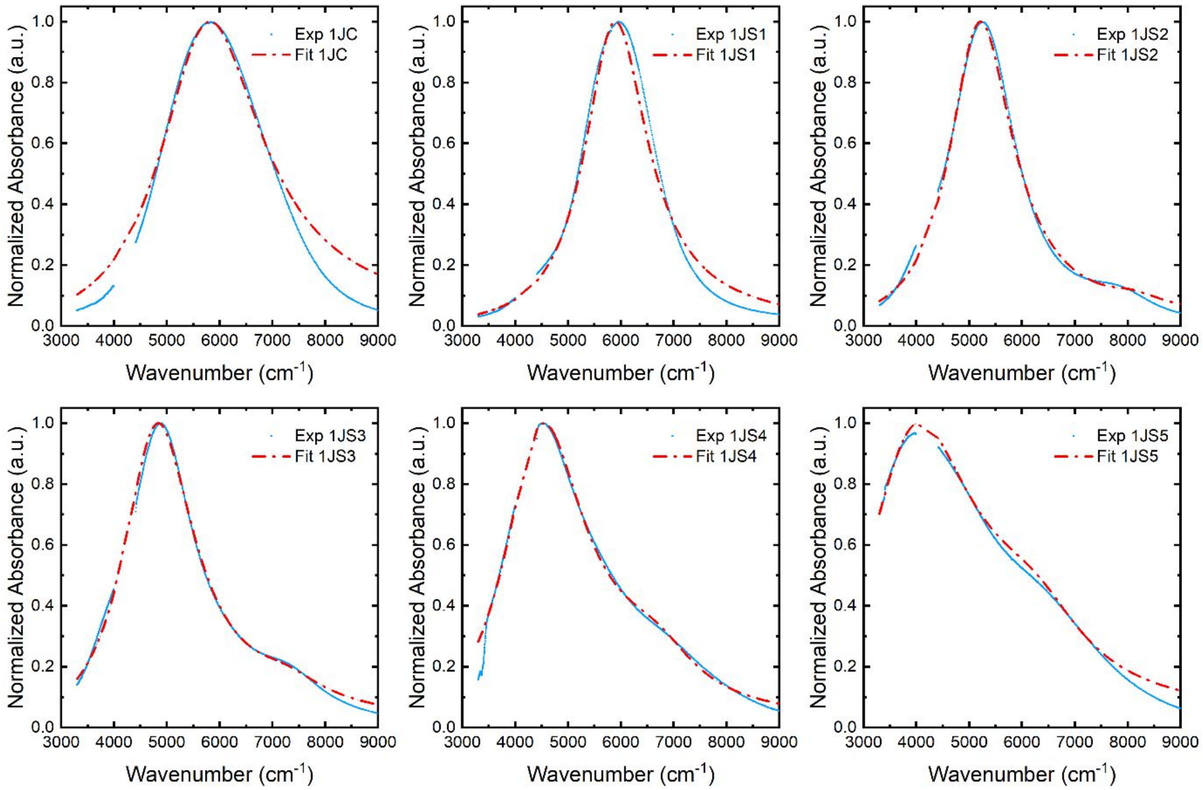

Figure S5. Experimental normalized absorption spectra (blue) and corresponding fitting (dashed-red)) obtained with 2-layer (core case) and 3-layer optical modeling for seed-mediated method. Main text corresponding spectra are found in Figure 1j.

Parameter	1JC0	1JS1	1JS2	1JS3	1JS4	1JS5
ω_{core} (cm^{-1})	17901.364	19901.313	18521.023	17736.340	17441.628	16710.759
ω_{shell} (cm^{-1})	1	18966.670	15600.148	14011.601	12799.765	11730.904
ne_{core} (m^{-3})	1.429E27	1.767E27	1.530E27	1.403E27	1.357E27	1.246E27
ne_{shell} (m^{-3})	4.460E18	1.605E27	1.085E27	8.757E26	7.308E26	6.138E26
γ_{core}(cm^{-1})	2366.9194	2164.1757	1636.4460	1604.9372	1325.9771	1661.4513
γ_{shell} (cm^{-1})	100000.05	1135.4208	1237.8475	1718.2789	2353.5744	2999.9753
mfp_{core}(nm)	2.26315	2.65625	3.34848	3.3171	3.97035	3.07952
mfp_{shell} (nm)	7.8278E-5	4.90317	3.94813	2.64772	1.8199	1.34713
N_{TOTAL}	301.98232	300.93886	312.60837	323.36046	324.42413	313.76636
N_{core}	301.98232	102.42588	218.41847	238.8461	231.74288	188.31531
N_{shell}	9.9093E-7	198.51298	94.18991	84.51436	92.68125	125.45104
R (nm)	4.25	5.15	6.3	7.25	7.65	8.65
$Core$ (nm)	3.69474	2.40102	3.24216	3.43804	3.44185	3.3048
$Shell$ (nm)	0	1.1126	0.55607	0.55526	0.69997	1.09007

$DL\ (nm)$	0.55526	1.63638	2.50177	3.2567	3.50818	4.25513

Table S2. Fitting parameters corresponding to core (C0) and shell (S1, S2, S3, S4, S5) samples obtained via seed-mediated synthesis method corresponding to Figure 1j.

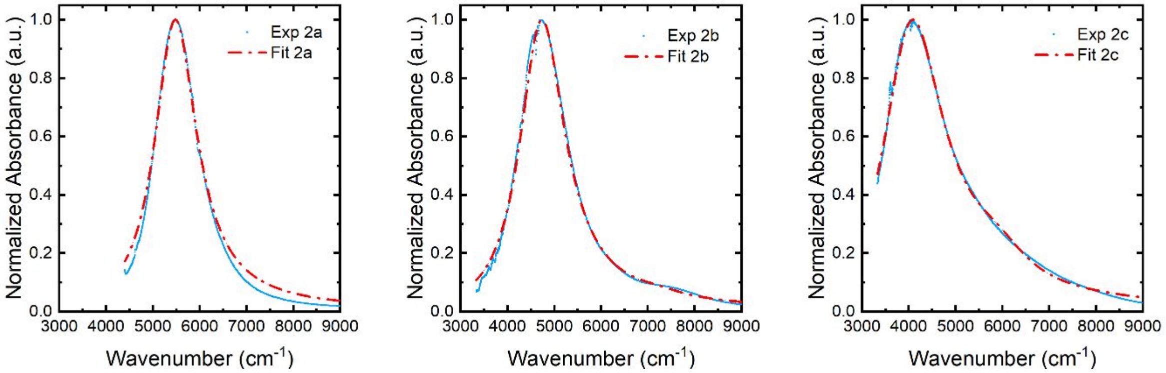

Figure S6. Experimental normalized absorption spectra (blue) and corresponding fitting (dashed-red)) obtained with 2-layer (core case) and 3-layer optical modeling for seed-mediated method with modified precursor amount and rate of injection. Main text corresponding spectra are found in Figure 2f.

Parameter	2a	2b	2c
ω_{core} (cm^{-1})	16899.686	15846.310	14631.806
ω_{shell} (cm^{-1})	1	14145.3262	11418.245
ne_{core} (m^{-3})	1.274E27	1.120E27	9.549E26
ne_{shell} (m^{-3})	4.460E18	8.925E26	5.815E26
γ_{core}(cm^{-1})	1104.88093	1135.85973	1275.71599
γ_{shell} (cm^{-1})	100000.03	1214.32289	1871.51109
mfp_{core}(nm)	4.66563	4.34778	3.67072
mfp_{shell} (nm)	7.82781E-5	3.77035	2.12087
N_{TOTAL}	368.82642	339.45573	330.12504
N_{core}	368.82642	154.88449	222.16837
N_{shell}	1.19274E-6	184.57123	107.95668
R (nm)	4.75	5.25	6.15
$Core$ (nm)	4.10396	3.20798	3.81543
$Shell$ (nm)	0	1.1433	0.82403
$DL\ (nm)$	0.64604	0.89872	1.51054

Table S3. Fitting parameters corresponding to core (2a) and shell (2b, 2c) samples obtained via seed-mediated synthesis method corresponding to Figure 2f.

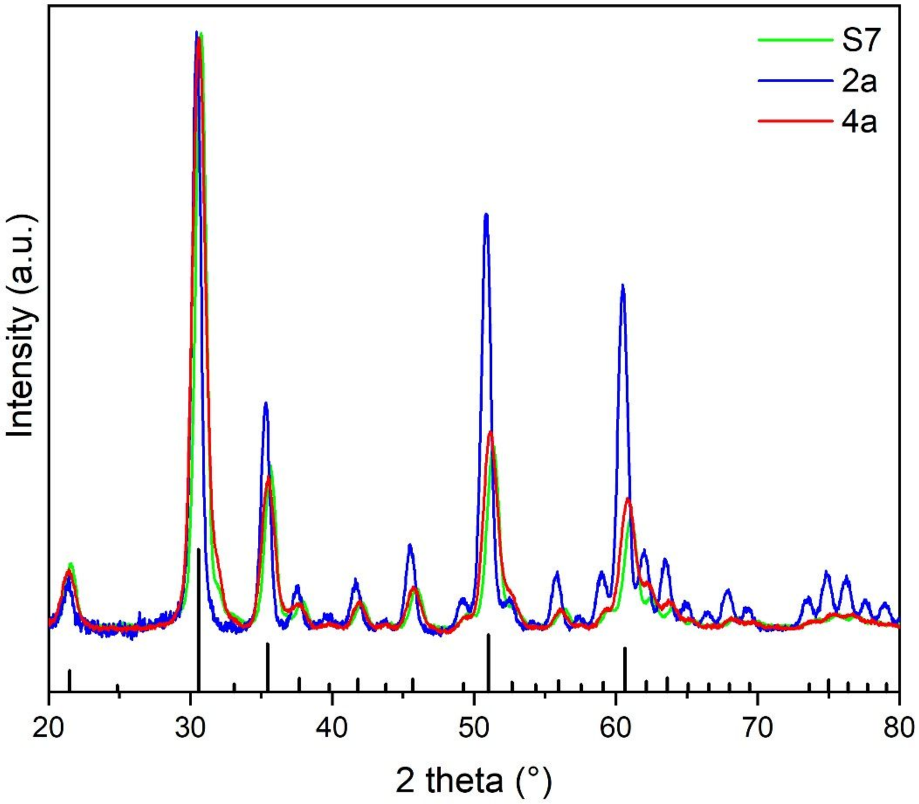

Figure S7. pXRD patterns of S7, 4a and 2a samples together with reflections of cubic In1.94O3Sn0.06 (black bars, ICSD number 050847).

a

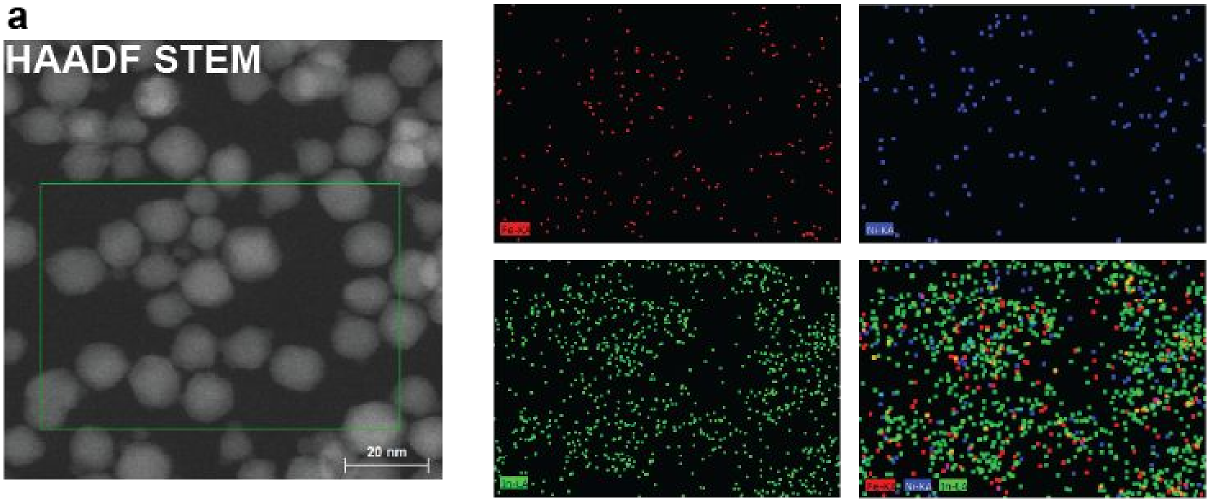

b

STEM-EDS analysis

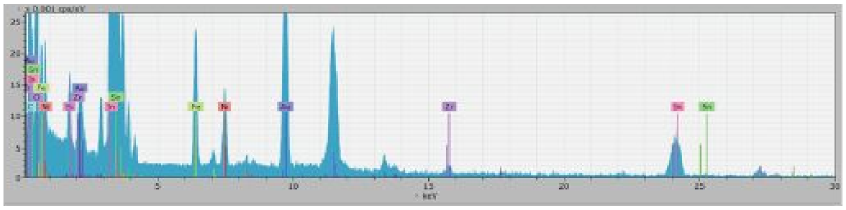

	Element	Series	Net	Atom C. [at.%]	Rel. error (1 Sigma) %
	In	K series	2575	79.81	3.73
	Fe	K series	2884	9.24	4.12
	Ni	K series	1727	5.81	4.73
	Sn	K series	140	5.13	10.19
	Au	L series	10125	0.00	
	C	K series	8045	0.00	
	Si	K series	849	0.00	
	O	K series	5471	0.00	
	Zr	K series	250	0.00	
	Ag	K series	182	0.00	
			Total	100.00	

Figure S8. (a). HAADF STEM images of 4a and elemental mapping for In (green), Fe (red), and Ni (blue). (b) STEM- EDS analysis for quantifying In, Fe, Sn and Ni over a large number of particles.

Samples	Sn/In	Fe/In	Ni/In
2a	0.047		
2b	0.022		
2c	0.008		
4a	0.023	0.14	0.0877

Figure S9. Elemental ratios of samples (2a-c, 4a) measured by ICP-OES.

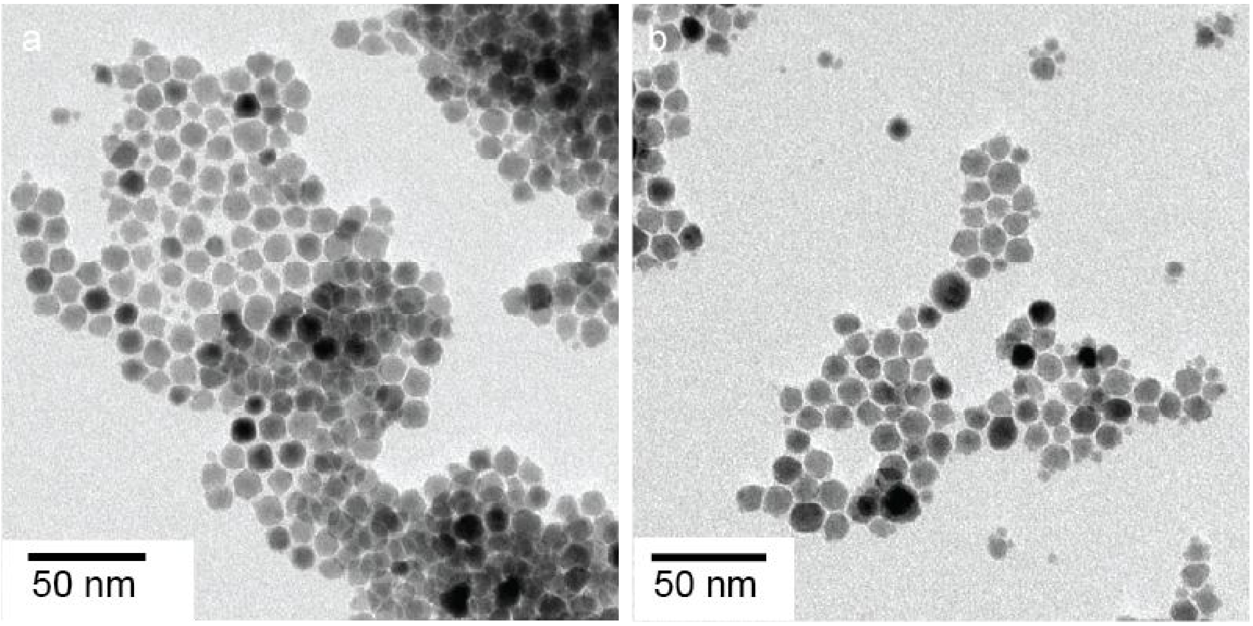

Figure S10. BF-TEM images (a) and (b) of the mixed nanocrystals formed during seeded core/shell indium tin oxide nanocrystals reacted with freshly prepared In/Fe oleate (3mmol, 3:2).

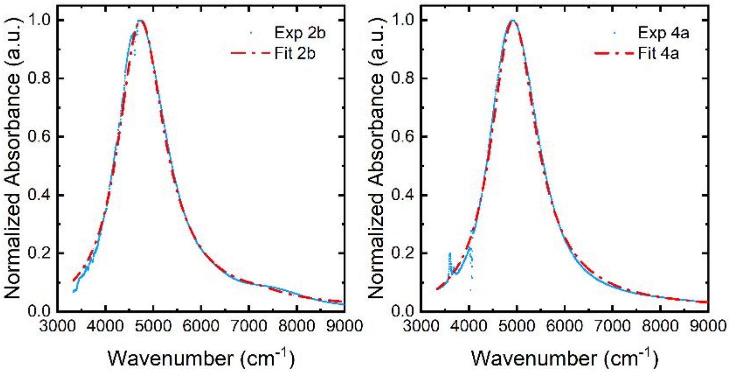

Figure S11. Experimental normalized absorption spectra (blue) and corresponding fitting (dashed-red)) obtained with 3-layer optical modeling comparing undoped In_2O_3 shelling and Fe/Ni shelling for seed-mediated method. Main text corresponding spectra are found in Figure 5a.

Parameter	2b	4a
ω_{core} (cm^{-1})	15846.310	14820.364
ω_{shell} (cm^{-1})	14145.3262	10064.462
ne_{core} (m^{-3})	1.120E27	9.79684E26
ne_{shell} (m^{-3})	8.925E26	4.51803E26
γ_{core}(cm^{-1})	1135.85973	1123.42066
γ_{shell} (cm^{-1})	1214.32289	1444.98002
mfp_{core}(nm)	4.34778	4.20407
mfp_{shell} (nm)	3.77035	2.52525
N_{TOTAL}	339.45573	384.929
N_{core}	154.88449	383.75778
N_{shell}	184.57123	1.17122
R (nm)	5.25	4.9
$Core$ (nm)	3.20798	4.53901
$Shell$ (nm)	1.1433	0.00999
DL (nm)	0.89872	0.351

Table S4. Fitting parameters corresponding to undoped In_2O_3 shelling (2b) and Fe/Ni shelling (4a) for seed-mediated method to Figure 2a.

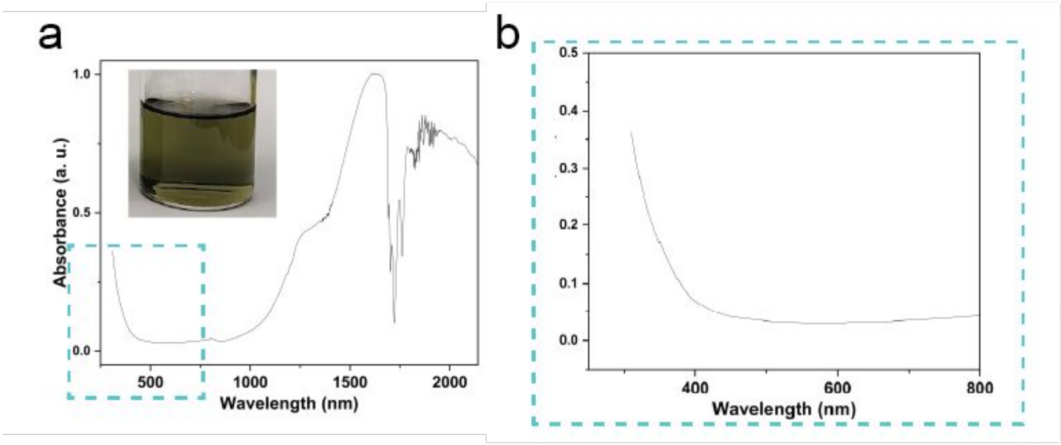

Figure S12. (a) UV–vis spectra of S13b (black line; olive hue dispersion in Toluene). (b) Expanded inset highlighting no a shoulder at ~480 nm.

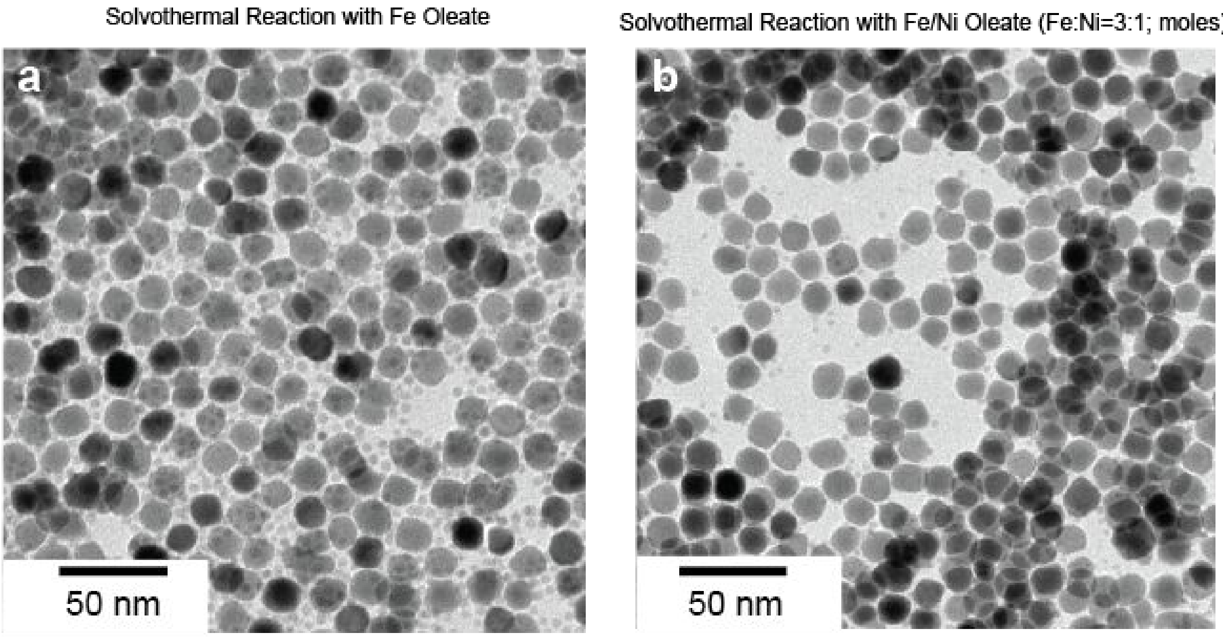

Figure S13. BF-TEM images (a) and (b) of the mixed nanocrystals formed during seeded core/shell indium tin oxide nanocrystals reacted with freshly prepared Fe oleate (3mmol) and Fe/Ni oleate (3mmol, 2:1), respectively. Neither precursor combination yields coherent shell formation, confirming the essential role of indium oleate in stabilizing the surface and enabling epitaxial shell growth.

References

(1) Petrini, N.; Ghini, M.; Curreli, N.; Kriegel, I. Optical Modeling of Plasmonic Nanoparticles with Electronically Depleted Layers. *The Journal of Physical Chemistry C* **2023**, *127* (3), 1576–1587. https://doi.org/10.1021/acs.jpcc.2c05582.

(2) Ghini, M.; Curreli, N.; Lodi, M. B.; Petrini, N.; Wang, M.; Prato, M.; Fanti, A.; Manna, L.; Kriegel, I. Control of Electronic Band Profiles through Depletion Layer Engineering in Core–Shell Nanocrystals. *Nat Commun* **2022**, *13* (1), 537. https://doi.org/10.1038/s41467-022-28140-y.

(3) Agrawal, A.; Kriegel, I.; Milliron, D. J. Shape-Dependent Field Enhancement and Plasmon Resonance of Oxide Nanocrystals. *The Journal of Physical Chemistry C* **2015**, *119* (11), 6227–6238. https://doi.org/10.1021/acs.jpcc.5b01648.
